# Molecular Mechanisms Controlled by mTOR in Male Reproductive System

**DOI:** 10.3390/ijms20071633

**Published:** 2019-04-02

**Authors:** Bruno P. Moreira, Pedro F. Oliveira, Marco G. Alves

**Affiliations:** 1 Department of Microscopy, Laboratory of Cell Biology, Institute of Biomedical Sciences Abel Salazar (ICBAS) and Unit for Multidisciplinary Research in Biomedicine (UMIB), University of Porto, 4050-313 Porto, Portugal; brunommoreira9@gmail.com (B.P.M.); pfobox@gmail.com (P.F.O.); 2 i3S-Instituto de Investigação e Inovação em Saúde, University of Porto, 4200-135 Porto, Portugal; 3 Department of Genetics, Faculty of Medicine, University of Porto, 4200-450 Porto, Portugal

**Keywords:** mTOR, spermatogenesis, male fertility, Sertoli cells

## Abstract

In recent years, the mammalian target of rapamycin (mTOR) has emerged as a master integrator of upstream inputs, such as amino acids, growth factors and insulin availability, energy status and many others. The integration of these signals promotes a response through several downstream effectors that regulate protein synthesis, glucose metabolism and cytoskeleton organization, among others. All these biological processes are essential for male fertility, thus it is not surprising that novel molecular mechanisms controlled by mTOR in the male reproductive tract have been described. Indeed, since the first clinical evidence showed that men taking rapamycin were infertile, several studies have evidenced distinct roles for mTOR in spermatogenesis. However, there is a lack of consensus whether mTOR inhibition, which remains the experimental approach that originates the majority of available data, has a negative or positive impact on male reproductive health. Herein we discuss the latest findings concerning mTOR activity in testes, particularly its role on spermatogonial stem cell (SSC) maintenance and differentiation, as well as in the physiology of Sertoli cells (SCs), responsible for blood–testis barrier maintenance/restructuring and the nutritional support of spermatogenesis. Taken together, these recent advances highlight a crucial role for mTOR in determining the male reproductive potential.

## 1. Introduction

Homeostasis, a term coined by Walter Bradford Cannon [1], represents the state of internal conditions of an organism where the equilibrium for optimal functioning is achieved. This equilibrium is constantly being threatened by internal and external stimuli which can compromise key processes including cell growth, proliferation and apoptosis, therefore compromising biological homeostasis. These processes are regulated by several factors including nutrients and hormones, which trigger complex signaling pathways. One of these pathways, involving the mammalian target of rapamycin (mTOR), has emerged in the last decade as a central regulator of biological homeostasis, being associated with protein synthesis, glucose metabolism and cytoskeleton organization among other functions [2,3]. mTOR is a well conserved Ser/Thr protein kinase of approximately 290 kDa, which was originally identified in yeast but is present in all mammalian and non-mammalian cells integrating cellular energy status, thus regulating cellular metabolism [3]. This kinase exists in two functionally and structurally distinct forms depending on the proteins that associate with the core component: The mTOR complex 1 (mTORC1) and the mTOR complex 2 (mTORC2) [4,5,6]. Interestingly, both complexes present different sensitivities to mTOR inhibitors [7,8]. As a consequence of being so versatile, the study of mTOR has a high degree of complexity.

In recent years, mTOR has been associated with spermatogenesis. Studies have demonstrated that mTOR controls glucose consumption and redox balance in Sertoli cells (SCs), highlighting a direct involvement for this pathway in the nutritional support of spermatogenesis [9]. Furthermore, mTOR is also involved in the maintenance and restructuring of the blood–testis barrier (BTB), a key event in the seminiferous epithelium cycle [10,11,12].

Notably, mTOR is intimately linked with eukaryotic cell growth and metabolism, regulating these processes according to several environmental inputs [3]. Metabolism is known to be pivotal to spermatogenesis [13] as it is responsible for the formation of spermatozoa and thus is directly associated with the fertility potential of an individual. This is highlighted in the seminiferous tubule epithelium across the different stages that are classified according to the development stage of germ cells and their association with SCs [14]. The somatic SCs have key roles for the success of spermatogenesis as they are responsible for the physical and nutritional support of germ cells. The metabolic cooperation established between Sertoli and germ cells is essential, since germ cells cannot use glucose and rely on SCs production of lactate to satisfy their metabolic needs [15,16]. Adjacent SCs establish the BTB, an immune-privileged environment, where germ cells safely develop from the attack of immune system cells [17]. During spermatogenesis, BTB is reorganized to allow the transport of germ cells to the lumen of the seminiferous tubules, where one of the last steps of spermatogenesis occurs. This complex network of steps and checkpoints is tightly coordinated to ensure that no disruption occurs, which could lead to infertility. In the last decade, several studies were focused on these two steps and how mTOR regulates them, which revealed new clues into the molecular and biochemical mechanisms behind mTOR pathway and male fertility [9,10,11,12,18,19]. Herein, we do a follow up concerning the most recent studies focused on mTOR and male reproduction, which revealed new clues in the everlasting puzzle of mTOR as a central regulator of spermatogenesis, and hence male fertility.

## 2. mTOR Signaling and Cell Physiology: Brief Overview

Life began billions of years ago with the appearance of unicellular organisms [20]. These simple life forms satisfy their metabolic needs according to the availability of nutrients [21]. Fast-forwarding in time, these organisms evolved into pluricellular organisms, which are composed of millions of cells, each one with a specific purpose [22]. These organisms react accordingly to external stimuli, that is, they have the ability to adapt their metabolic needs to the situation [21]. This is only possibly due to the existence of metabolic pathways that can integrate the information and react accordingly. mTOR plays a central role in the signaling network that balances the metabolic signals of growth factors, energy status, oxygen, stress and amino acids, and outputs the correct cascade of events resulting in protein and lipids synthesis or autophagy, accordingly to the stimuli [3].

**Figure 1:**
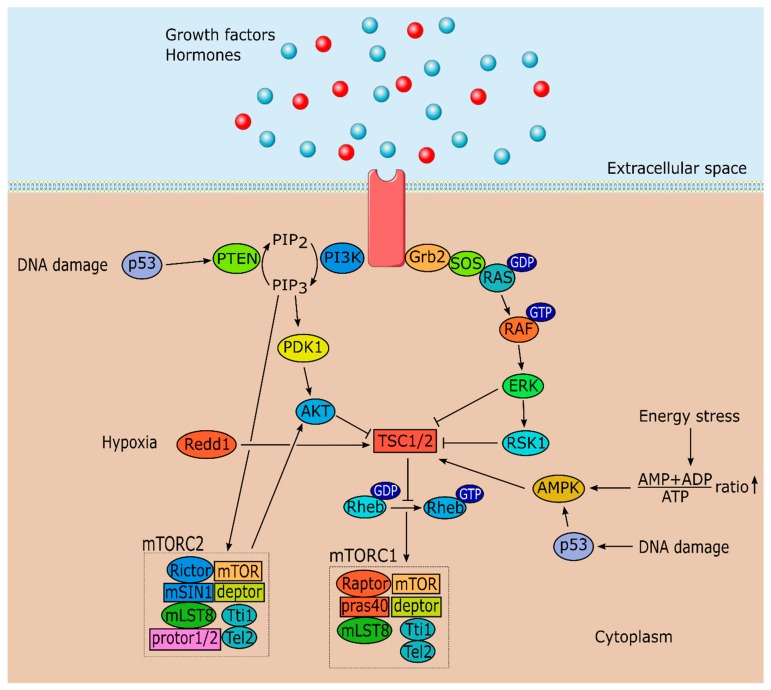
Schematic illustration of rapamycin (mTOR) signaling pathway. mTOR forms two functional complexes, mTORC1 and mTORC2 which are involved in different physiological processes. mTORC1 is regulated by growth factors/hormones, DNA damage, energy status and oxygen levels. mTORC2 is also regulated by growth factors and is involved in AKT phosphorylation. Abbreviations: AKT: protein kinase B; AMPK: AMP-activated protein kinase; deptor: DEP (Dishevelled, Egl-10 and Pleckstrin) domain-containing mTOR-interacting protein; ERK: Extracellular signal regulated kinase; Grb2: Growth factor receptor bound protein 2; mLST8: Mammalian lethal with sec-13 protein 8; mSIN1: Mammalian stress-activated protein kinase interacting protein; p53: Cellular tumor antigen p53; PDK1: 3-phosphoinositide-dependent protein kinase-1; PI3K: Phosphoinositide 3-kinase; pras40: Proline-rich Akt substrate 40 kDa; protor1/2: Protein observed with rictor 1 and 2; PTEN: Phosphatase and tensin homolog; raptor: Regulatory associated protein of mTOR; Redd1: Protein regulated in development and DNA damage response 1; Rheb: Ras homolog enriched in brain GTPase; rictor: rapamycin insensitive companion of mTOR; RSK1: p90 ribosomal S6 kinase 1; SOS: Ras-guanine exchange factor; TSC1/2: Tuberous sclerosis 1/2. 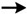 stimulation. 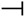 inhibition.

mTOR can form two functionally and structurally distinct forms, depending on the associated proteins. mTORC1 is formed by mTOR, regulatory associated protein of mTOR (raptor), proline-rich Akt substrate 40 kDa (pras40), DEP (Dishevelled, Egl-10 and Pleckstrin) domain-containing mTOR-interacting protein (deptor), mammalian lethal with sec-13 protein 8 (mLST8) and the Tti1/Tel2 complex (Figure 1) [5,23,24,25,26,27]. Although mTORC2 shares several protein components with mTORC1 including mTOR, deptor, mLST8 and the Tti1/Tel2 complex; it is composed by rapamycin insensitive companion of mTOR (rictor), mammalian stress-activated protein kinase interacting protein (mSIN1) and protein observed with rictor 1 and 2 (protor1/2) (Figure 1) [6,28,29]. Thus, mTORC1 or mTORC2 are formed depending on whether raptor or rictor associate with the core component. Nonetheless, there is still much to be discovered concerning mTOR complex proteins and how they interact with mTOR structure and signaling.

mTORC1 is considered the rapamycin-sensitive complex [8] while mTORC2 was usually known as the rapamycin-insensitive complex [7]. This concept has changed since mTORC2 assembly was shown to be inhibited by long term rapamycin treatment in some cell types [30]. This probably occurs due to the inability of mTOR bounded to rapamycin to associate with rictor, therefore impairing the formation of new mTORC2 complexes. Thus, as the name easily suggests, mTOR is referred as the mammalian target of rapamycin, a 31-membered macrocyclic natural product produced by several actinomycetes. Interestingly, rapamycin was found in a screening for anti-fungal agents [31]. Besides antifungal properties, rapamycin also has immunosuppressive, antitumoral and lifespan extension properties, which turned this molecule into a desired tool to study cell growth, and lately to be used as a potential tool to fight metabolic diseases [32,33,34]. Rapamycin inhibits mTORC1 by associating with its intracellular receptor FK506-binding protein 12 (FKBP12) forming a gain of function complex which interacts with the corresponding FKBP12–rapamycin binding domain located in mTOR, inhibiting mTOR activity by occluding substrates from the active site [8].

Tuberous sclerosis complex (TSC1/2), formed by TSC1 and TSC2, a GTPase-activating protein, functions as an upstream regulator of mTORC1, converting the Ras homolog enriched in brain GTPase (Rheb) into its inactive GDP bound state (Figure 1) [35,36]. This conversion blocks Rheb from stimulating mTORC1 kinase activity. mTORC1 kinase activity can be triggered by several stimuli such as: Growth factors via the IRS-PI3K and MAPK/ERK pathways; the energy status of the cell (ATP/AMP ratio) and DNA damage via AMP-activated protein kinase (AMPK) pathway; certain stresses including oxygen levels; and nutrient status via amino acids that function as sensors (Figure 1). Nutrient level detection by mTOR is the least described mechanism, although progress has been made in recent years [37,38]. These inputs, excluding nutrient level detection, exert their action on mTOR through modulation of TSC1/2 activity (Figure 1) [39]. As their name suggests, mutations on TSC1/2 originate tuberous sclerosis, a disease characterized by the development of hamartomas (mostly benign malformations) in multiple organ systems [40]. Stimulation by growth factors (e.g., insulin and IGF-1) activates PI3K and MAPK pathways, which results in the phosphorylation of TSC1/2 by protein kinase B (Akt), by p90 ribosomal S6 kinase 1 (RSK1) and by extracellular signal regulated kinase (ERK) (Figure 1) [41,42,43,44,45]. This phosphorylation inactivates TSC1/2, which results in mTORC1 activation.

mTORC1 is also involved in the response to stress signals such as low energy levels. AMPK, a vital enzyme that functions as an intracellular energy sensor, phosphorylates TSC1/2 in these cases increasing TSC1/2 activity culminating in the reduction of mTORC1 kinase activity (Figure 1) [46]. DNA damage signals are also regulated by mTORC1 activity. In these cases, p53 dependent transcription activates AMPK resulting in mTOR inhibition (Figure 1) [47,48]. Another mechanism involved in the response to stress signals is phosphatase and tensin homolog (PTEN) activation mediated by p53, which downregulates the entire PI3K-Akt-mTORC1 axis culminating in autophagy (Figure 1) [47,49]. TSC1/2 can also be directly activated by protein regulated in development and DNA damage response 1 (Redd1) in hypoxia situations which inhibits mTOR (Figure 1) [50,51].

Concerning mTORC2, less information is known about this complex signaling pathways and its upstream and downstream regulators. Nonetheless, studies have shown mSIN1 is required for mTORC2 assembly and kinase activity [52], which in turn activates Akt (Figure 1), and serum and glucocorticoid-regulated kinase 1 [53,54]. Moreover, under non-stimulated conditions, pleckstrin homology (PH) domain of mSIN1 interacts with mTOR kinase domain to suppress mTORC2 activity. However, upon stimulation by insulin, activated PI3K forms PtdIns(3,4,5)P_3_, which interacts with PH-mSIN1 to release its inhibition on mTOR kinase domain, leading to mTORC2 activation (Figure 1) [55]. This activation results in phosphorylation of Akt at the hydrophobic motif of Ser^473^ setting in motion a cascade of phosphorylation by other proteins until Akt is fully activated creating a positive feedback loop between Akt and mTORC2 [56]. Interestingly, while TSC1/2 inhibits mTORC1, it can activate and associate with mTORC2 [57]. Another mechanism suggests that mTORC2 associates with ribosomes in a growth factor sensitive manner and these ribosomes are necessary for mTORC2 kinase activity [58]. Furthermore, the rapamycin insensitive complex also modulates the phosphorylation of several members of the protein kinase C (PKC) involved in the regulation of the actin cytoskeleton and cell migration [6,7].

As referred above, mTOR complexes are different per se. Besides structural differences, they also have different sensitivities to rapamycin and different upstream and downstream outputs. mTORC1 integrates signals from several sources, including growth factors, stress signals and amino acids status, and responds accordingly, regulating cell growth by promoting protein and lipids synthesis, ribosomes biogenesis, cell metabolism and ATP production. mTORC1 also has a key role in inhibiting autophagy. Concerning mTORC2, it is involved in cell proliferation, surveillance, metabolism and cytoskeleton organization, mainly through Akt, which phosphorylates downstream targets positively regulating these processes.

## 3. mTOR and Male Fertility: Evidence from Testis Signaling

**Figure 2:**
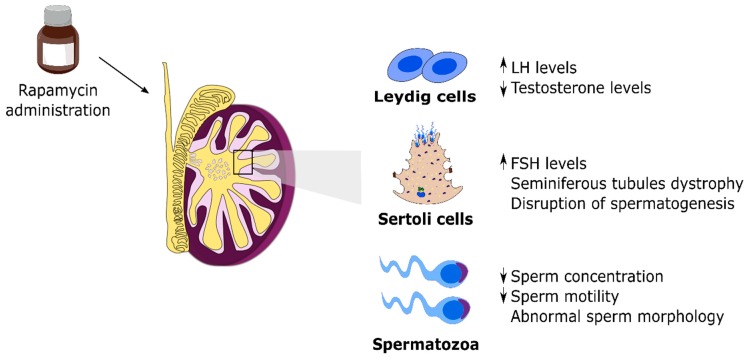
Effects of mTORC1 inhibitor (rapamycin) administration on the testicular function and sperm production. The figure depicts the outcomes of several clinical studies where rapamycin was used as an immunosuppressant which resulted in male infertility. Posterior studies using mice models exposed to rapamycin revealed the deleterious effects of this compound to testicular morphology, gonadotropins and testosterone levels, and overall for spermatogenesis.

mTOR is regarded as the central integrator of several signals, as listed above, regulating metabolism, cellular growth and proliferation. However, little information concerning mTOR and its functions was known just a couple of decades ago. This paradigm has shifted and mTOR has been a target of great scientific interest in recent years. This outburst of information occurred due to the use of mTOR inhibitors in several works with clinical intentions [59,60,61,62]. Currently, mTOR inhibitors are still a target of several studies with the aim to be used as pharmacological agents in the treatment of diseases, including cancer and diabetes [63,64,65]. These studies paved the way to outline mTOR signaling pathway and functions, although there is still much to be done. Thus, most of the information gathered concerning mTOR is due to the use of rapamycin. As mentioned before, rapamycin, also known as sirolimus, is an allosteric inhibitor of mTOR, approved in 1999 by the Food and Drug Administration under the commercial name of ^®^Rapamune to be used as an immunosuppressant preventing organ rejection in transplants [66]. Although rapamycin fulfilled its purpose, several side effects emerged from its use. Male infertility was one of the most striking side effects described in patients after few years of rapamycin use [67]. Specifically, the most relevant reported effects were low sperm count, decreased motility and decreased sperm vitality as well as negative impact on sexual hormone levels and lower rate of fathered pregnancies when compared with individuals treated with other immunosuppressants (Figure 2) [68,69,70]. These were the first studies that provided evidence for a negative effect of prolonged rapamycin use on male fertility. Subsequent studies were more focused on the root responsible for the impaired fertility parameters reported and, using mice models, revealed that mTOR inhibitors, particularly rapamycin, induced major histological changes in testicular structure followed by impairment of testicular development and of spermatogenesis (Figure 2) [71,72]. Overall, rapamycin was clearly demonstrated to be capable of inducing testicular toxicity. However, those effects mediated by rapamycin were shown to be reversible. Switching from a rapamycin-based therapy to another immunosuppressant recovered normal fertility parameters and sexual hormone levels, thus restoring the fertility of men previously treated with rapamycin [72,73]. Nevertheless, the mechanisms through which mTOR inhibitors induce infertility remain largely unknown.

**Figure 3:**
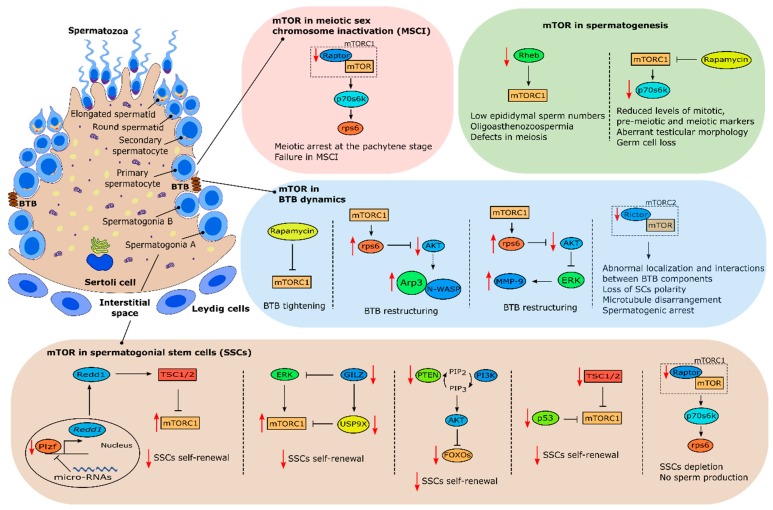
Involvement of mTOR in several processes linked with male fertility. mTORC1 is required for a correct meiotic sex chromosome inactivation. Furthermore, mTOR inhibition of mTORC1 or knockdown of Rheb results in germ cell loss, reduced epididymal sperm numbers, defects in testicular morphology and impairment of meiosis. mTOR is also directly involved in BTB dynamics, with mTORC1 promoting BTB restructuring and mTORC2 promoting BTB maintenance. mTOR inhibition is also required for spermatogonial stem cell (SSC) self-renewal. However, knockdown of raptor impairs spermatogenesis which shows that mTORC1 presence is required for SSCs self-renewal and a balance must occur between mTOR inhibition and mTOR activation for a correct SSCs proliferation and differentiation. Abbreviations: BTB: Blood–testis barrier; AKT: Protein kinase B; ERK: Extracellular signal regulated kinase; FOXOs: Forkhead box proteins; GILZ: Glucocorticoid-induced leucine zipper; MMP-9: Matrix metallopeptidase 9; MSCI: Meiotic sex chromosome inactivation; mTOR: Mammalian target of rapamycin; N-WASP: Neuronal Wiskott–Aldrich syndrome protein; p53: Cellular tumor antigen p53; p70s6k: p70S6 kinase; PI3K: Phosphoinositide 3-kinase; Plzf: Promyelocytic leukaemia zinc finger; PTEN: Phosphatase and tensin homolog; raptor: Regulatory associated protein of mTOR; Redd1: Protein regulated in development and DNA damage response 1; Rheb: Ras homolog enriched in brain GTPase; rictor: Rapamycin insensitive companion of mTOR; rps6: Ribosomal protein S6; TSC1/2: Tuberous sclerosis complex; USP9X: Spermatogonial deubiquitinase probable ubiquitin carboxyl-terminal hydrolase FAF-X. 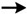 stimulation. 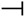 inhibition. 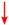 downregulation/knockout. 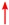 upregulation.

In 2010, Hobbs et al., showed that mTOR plays an important role in spermatogonial stem cell (SSC) maintenance [74]. For clarification, in this review, SSCs will be used to define undifferentiated germline cells that can self-renewal. It was shown that mice lacking promyelocytic leukaemia zinc finger (Plzf) (*Plzf*^−/−^), a transcription factor essential for SSCs maintenance (Figure 3) [75,76], presented aberrant mTORC1 activity which inhibited SSC response to glial cell-derived neurotrophic factor (GNDF), a known growth factor required for SSC self-renewal, through negative feedback. *Plzf* ^−/−^ mice mTORC1 hyperactivity was due to lack of Plzf-mediated Redd1 transcriptional activity which inhibits mTORC1 (Figure 3) [74]. Interestingly, a recent study by Daguia Zambe et al., suggested that Plzf inhibition of mTOR was regulated by micro-RNAs, specifically miR-19b-3p, opening new exciting possibilities to further understand mTOR’s role in SSC maintenance [77]. Other study has suggested that conditional knockout of *FOXO* (forkhead box protein O) *1, FOXO3* and *FOXO4*, Akt-regulated factors involved in stem-cell renewal [78], within the germ line-specific Vasa-Cre blocks SSCs self-renewal and differentiation [79]. Conditional knockout of *PTEN* also phenocopied FOXOs conditional inactivation phenotype suggesting that PI3K-Akt signaling and Akt inhibition of FOXOs are involved in the homeostasis of SSCs proliferation and differentiation (Figure 3) [79]. Interestingly, similar results were obtained with conditional knockout of *PTEN* in hematopoietic stem cells, a phenotype that could be partially rescued by rapamycin [80]. *PTEN* conditional inactivation should result in mTOR activation which would explain why rapamycin treatment restored hematopoietic stem cells self-renewal ability. Logically, conditional inactivation of PTEN in germ cells should result in Akt-stimulated mTOR activation further corroborating the results described by Hobbs et al., evidencing the role of mTOR in SSCs maintenance and differentiation. Nevertheless, this remains to be confirmed.

p53, the well-recognized tumor suppressor agent, seems to be another agent involved in suppressing mTOR activity to allow for SSC self-renewal. Under genotoxic conditions, p53 induces cell-cycle arrest through inhibition of mTOR [81]. Although many studies were focused on p53 functions under these conditions, mounting evidence has suggested the involvement of p53 in the regulation of stem cell processes under normal physiological conditions [82]. Recently, *p53* knockout mice testes were shown to augment mTORC1 activity during early spermatogonial differentiation which induced exhaustion of the SSC pool, driving them out of the undifferentiated state, indicating that the p53-mTORC1 pathway is also involved in regulating the SSC differentiation process (Figure 3) [83]. Furthermore, recent studies in mice, where germ cell conditional knockouts were created for *TSC1* and *TSC2*, resulted in mTOR aberrant activity which induced spermatogonial differentiation depleting the SSC pool (Figure 3) [84,85]. Both studies reported lower testis weight and a higher percentage of degenerated seminiferous tubules when compared with controls which clearly highlights a role for mTOR in spermatogenesis. Interestingly, in those studies, mTOR activation was shown to be stage-dependent concerning spermatogonial development. Self-renewing stem cells had mTORC1 activity suppressed while progenitors committed to differentiation had mTORC1 activity induced, in both conditional knockout mice models [84,85]. Those findings clearly suggest a role for mTORC1 supervising and deciding stem cells fate.

Glucocorticoid-induced leucine zipper (GILZ), an essential factor for spermatogenesis [86,87], was also demonstrated to be an essential modulator of growth factor signaling in SCCs. Indeed, adult mice knockout for *GILZ* are characterized by SCCs exhaustion and germline degeneration [88]. *GILZ* knockout mice present aberrant mTORC1 activation, which was a downstream effect of aberrant activation of ERK/MAPK pathways (Figure 3) [88]. Treatment of these mice with Torin1, an mTOR inhibitor, rescued SSC depletion. Interestingly, expression of the spermatogonial deubiquitinase probable ubiquitin carboxyl-terminal hydrolase FAF-X (USP9X), an essential factor for a proper spermatogenesis [89], was also downregulated in *GILZ* knockout mice (Figure 3) [88]. Altogether, these data pinpoint exact mechanisms that help to explain how the decisions for the fate of SSCs are chosen. mTORC1 seems to be inhibited by GILZ through USP9X expression. GILZ also modulates mTORC1 through inhibition of upstream signals, including MAPK/ERK pathways which indicates that GILZ operates as an essential rheostat for growth factor signaling. In fact, Wang et al., demonstrated that mTORC1 balance between phosphorylated and inhibited states seems to be a key factor modulating SSCs fate. In that study, Wang and colleagues used an interesting approach to detect phosphorylated protein and phosphorylated sites after stimulation by GDNF, a growth factor required for SSC self-renewal [90,91]. This revealed that SSC proliferation is dependent on the GDNF/ERK modulation since the inhibition of this pathway impaired proliferation [92]. Interestingly, this process was dependent on mTORC1 phosphorylation, specifically in the Ser^863^ of mTORC1 component, raptor [92]. In vitro overexpression of this component resulted in an accelerated growth of SSCs while inhibition of raptor by deletion in mouse germline cells resulted in SSC depletion and impaired spermatogenesis. Taken together, these data validated previous studies and further expanded the knowledge on mTORC1 relevance in deciding the fate of SSCs. It seems that a specific raptor phosphorylation is required to decide the future of SSCs, and ERK pathway is involved. Indeed, two recent studies from Serra et al. focused on these issues and gave new insights on mTOR’s involvement in the fate of SSCs. Using two different germ cell knockout mice models of *mTOR* and *raptor* component respectively, these studies produced very interesting and surprising results. In the first study, germ cell knockout of *mTOR* core component (not the mTORC1 complex as a whole) resulted in no sperm production due to impairment of spermatogonial differentiation [93]. Interestingly, a small subset of SSCs remained in adult testes, indicating that mTOR is not required for the survival and maintenance of SSCs but rather for their proliferation and differentiation [93]. This phenotype clearly resembles the one reported by Busada et al., where inhibition of mTORC1 by rapamycin lead to impairment of spermatogonial differentiation [94]. This similarity suggests that mTOR effects on spermatogonial differentiation and proliferation are primarily mediated by mTORC1 and not mTORC2. In the second study, germ cell knockout of *raptor*, mTORC1’s core component, also resulted in no sperm production. However, interesting differences were observed comparatively to the first study. Spermatogonia from germ cell *raptor* knockout mice entered meiosis but were unable to complete it [95]. Interestingly, adult testes seminiferous tubules only had SCs due to SSC depletion [95]. These results clearly suggest that raptor is essential in the completion of meiosis and for the formation and maintenance of a fully functional pool of SSCs (Figure 3). Furthermore, unlike other studies where mTORC1 hyperactivation resulted in SSC differentiation but not a total depletion, the reported total depletion of the SSC pool could be attributed to inhibition of FOXOs, important factors in self-renewal of SSCs [79]. This could be due to a higher number of mTORC2 complexes being formed in response to the knockout of raptor. One of the well-known functions of mTORC2 is activation of Akt [54] which, as referred to above, is involved in the inhibition of FOXOs [79]. Nevertheless, this hypothesis remains to be fully tested and demonstrated.

Several other studies also showed that mTOR is heavily involved in spermatogenesis [96,97,98]. For instance, conditional knockout of *Rheb* in male germline resulted in oligoasthenoteratozoospermia and male infertility [96]. The authors could observe multiple defects in meiotic and post-meiotic stages of spermatogenesis, which resulted in an increase of sperm defects and overall severe reduction on epididymal sperm numbers (Figure 3) [96]. In addition, spermatid and spermatocytes production decreased with age while undifferentiated spermatogonia maintained the normal numbers, reflecting a delay in meiotic progression. Interestingly, Hobbs et al. previously observed that Rheb was not required for SSC self-renewal [74], but it seems that Rheb is crucial for meiotic progression. This is also a subject that deserves attention in years to come regarding mTOR and SSCs self-renewal and progression.

Retinoic acid is a key regulator of spermatogenesis, regulating spermatogonial differentiation via retinoic acid stimulated gene 8 (*STRA8*), a gene expressed in SSCs and preleptotene spermatocytes [99,100]. *STRA8* was shown to be necessary for differentiating spermatogonia to undergo morphological changes that define meiotic prophase and for these cells to exhibit the molecular hallmarks of meiotic chromosome cohesion, synapsis and recombination. In fact, male mice lacking *STRA8* gene function fail to enter meiotic prophase [101]. Sahin et al. confirmed that SSCs and preleptotene spermatocytes express several downstream effectors of the mTOR pathway including mTOR, p-mTOR, p70s6k, phosphorylated p70S6 kinase (p-p70s6k) and phosphorylated eukaryotic initiation factor 4E binding protein 1 (p-4E-BP1) [102]. Interestingly, inhibition of mTOR by rapamycin using cultured seminiferous tubules decreased the levels of p-p70s6k and p-4E-BP1, and also decreased the levels of proliferating cell nuclear antigen (PCNA) and STRA8, markers for proliferation and differentiation, respectively [102]. This clearly indicates that mTOR signaling is involved in the differentiation and stimulation of meiotic initiation of undifferentiated spermatogonia. A further study by this team aimed to unveil mTOR’s role in meiotic initiation and progression during post-natal development, specifically in the first wave of spermatogenesis, and in the adult mice. Administration of rapamycin in post-natal testes decreased p-p70s6k and STRA8 levels while STRA8 levels were increased after administration of retinoic acid, as expected [97]. Interestingly, administration of rapamycin during four weeks in adult testes induced germ cell loss, disorganization of testicular morphology and vacuolization (Figure 3). Furthermore, the levels of STRA8 and DNA meiotic recombinase 1 (Dmc1), a meiotic marker, were decreased [97]. Overall, mTOR signaling seems to be involved in the meiotic progression of spermatogenesis during not only the first wave of spermatogenesis but also in adult testes. Recently, Xu et al. demonstrated that mTOR and its downstream effectors are positively correlated with spermatogenesis at different development stages [98]. Interestingly, phosphorylated levels of p70s6k, ribosomal protein S6 (rps6) and 4E-BP1 were also gradually downregulated with age which could explain the decrease in male fertility potential that occurs as a consequence of aging. Inhibition of mTOR signaling by rapamycin decreased sperm number and downregulated protein levels of the phosphorylated effectors of mTOR referred above, except 4E-BP1 [98]. Interestingly, treatment with a PI3K inhibitor downregulated phosphorylated levels of 4E-BP1 suggesting that PI3K regulates this protein [98]. Overall, we can conclude that mTOR plays an important role in spermatogenesis by regulating this process through p70s6k activation.

In recent years, mTOR is also being closely related with meiotic sex chromosome inactivation (MSCI). MSCI is a process that, as the name suggests, occurs during the meiotic phase of spermatogenesis. In short, at the pachytene stage, transcriptional silencing of the male X and Y chromosomes occurs after autosomal chromosomes have completed pairing [103]. X and Y chromosomes are compartmentalized into a peripheral nuclear subdomain known as the XY body. Following meiosis II, when haploid daughter cells are formed, X and Y chromosomes are thought to be repressed until the end of spermatogenesis, although this is still a matter of debate [103]. Thus, MSCI is crucial for male fertility, as mutant mice with defects in MSCI are infertile due to meiotic arrest in prophase I [104,105]. A study by Xiong et al. revealed that raptor is an essential mTORC1 component for a correct MSCI and consequently, a correct meiosis. Mice with conditional knockout of *raptor* were sterile and had increased numbers of SSCs [106]. Furthermore, these mice exhibited meiotic arrest at the pachytene stage and XY chromosome were not repressed which suggests that mTORC1 is crucial for MSCI (Figure 3). MSCI failure was due to lower accumulation of ATR, a key mediator of meiotic silencing which is required to induce repressive epigenetic modifications on sex chromatin in pachytene spermatocytes [106]. On the contrary, another study has shown that meiotic progression and recruitment of silencing factors to sex chromosomes was normal in testes with conditional knockout of mTORC2 component *rictor* [107]. Overall, these reports suggest that rapamycin-mediated defects in meiosis and MSCI are mTORC1-dependent. In another study, inhibition of mTORC1 by chronic rapamycin treatment also caused defects in MSCI resulting in spermatogenic arrest. Recruitment of the essential silencing factor ATR to the chromatin was attenuated in the pachytene stage [108]. Interestingly, the rapamycin inhibitory effect was reversible following treatment withdrawal. Furthermore, rapamycin treated mice had a reduction in pachytene piRNA populations, suggesting that mTOR is involved in the homeostasis of noncoding RNA [108].

## 4. mTOR Pathway in Sertoli Cells and Male Fertility

SCs are unique polarized mesoepithelial cells responsible for the seminiferous tubules structure [109]. Extending from the basement membrane to the lumen of the seminiferous tubule, these cells are responsible for a panoply of functions, ranging from nourishment and structural support of developing germ cells, integration of upstream signals and secretion of factors and hormones accordingly, phagocytic activity of defective spermatogenic cells and the control of the microenvironment responsible for the correct development of germ cells [13,110]. SCs are known as “nurse cells” as they babysit germ cells through the different stages of spermatogenesis. In fact, SC extensions are in direct and permanent contact with germ cells to ensure their correct development. During spermatogenesis, germ cells must cross the seminiferous tubule to reach the border where spermiation is completed [111]. SC extensions and their microtubular network guide germ cells during this process. Finally, adjacent SCs establish the BTB, an immunoprivileged environment, restricting access by the immune system to these cells which could be identified as foreign agents by the immune system [17]. Structurally, BTB is composed by tight junctions, basal ectoplasmic specializations, desmosomes and gap junctions [17]. Those junctions are connected to the actin cytoskeleton and possess packed actin filament bundles that lie perpendicularly, connecting each adjacent SC through the plasma membrane [112]. These actin filament bundles are also enclosed by the endoplasmic reticulum cisternae giving BTB a remarkable strength and adaptability. In addition, BTB divides the seminiferous epithelium into two functionally and anatomically distinct compartments: 1) The basal compartment where SSCs and preleptotene spermatocytes reside not protected by the BTB; 2) the adluminal compartment where both meiosis and post-meiotic development occurs under the protection of the BTB [113]. Logically, this division suggests that developing spermatocytes must cross the BTB barrier to reach the lumen in order to fulfill the last steps of spermatogenesis. Preleptotene spermatocytes are the only germ cells transported across the BTB in different seminiferous epithelium stages according to the species (rat, mouse or human) [14,114]. Interestingly, this transport takes place quite rapidly, which suggests the existence of a tight and complex network regulating BTB modulation. The existence of a BTB, designated as old, which then gives origin to another BTB, designated as new, was initially pointed as the main mechanism. This was named as the intermediate compartment, in an attempt to explain this phenomenon [115]. This view has changed, and several important studies have shed new light on this topic.

Several studies have suggested that BTB remodeling is regulated, at least in part, by mTORC1 and mTORC2 (specifically by their particular subunits, raptor and rictor, respectively) [10,11,12,18]. This pathway targets several actin-regulating proteins which causes the cyclic reorganization of the F-actin network, remodeling the BTB. Several studies have shown a stage-specific expression of mTORC1 and mTORC2 subunits and downstream effectors (raptor/p-rps6 and rictor, respectively) during the epithelial cycle with the first being predominantly expressed at later stages of the seminiferous epithelium cycle and virtually undetectable in other stages while rictor expression is predominant in earlier stages of the epithelial cycle [10,11,12]. This expression pattern suggests that mTORC1 and mTORC2 have opposing effects in BTB dynamics and remodeling. In fact, it was reported that mTORC1 pathway promotes BTB remodeling, which causes this barrier to be “leaky”. Several studies using in vitro and in vivo approaches reported that inhibiting mTORC1 signaling, either by knockdown of *rps6* using RNAi or by rapamycin administration, promoted SCs tight junction permeability barrier effectively turning BTB “tighter” (Figure 3) [19]. In those studies, stage-specific p-rps6 expression in the BTB was colocalized with several putative BTB proteins including zonula occludens-1 (ZO-1) (adaptor protein connecting tight junctions to actin cytoskeleton), N-cadherin (a basal endoplasmic specialization protein), Arp3 (a component of the Arp2/3 complex at the BTB involved in changing the conformation of the actin network) and F-actin suggesting an involvement of p-rps6 in BTB remodeling in order to facilitate preleptotene spermatocytes transit to the adluminal compartment [19]. Other studies in mice with a constitutively active quadruple phosphomimetic mutant p-rps6 reported that this turns the BTB “leaky”, due to changes in F-actin bundle organization [10,11]. These studies also identified two pathways through which mTORC1 regulates BTB dynamics, the prpS6/Akt/Arp3/N-WASP and the p-rps6/Akt/MMP-9 pathways (Figure 3). In the first, alterations in the organization of actin microfilaments and in actin bundling activity destabilized BTB dynamics and SC tight junction barrier function [10]. These changes were caused by the rps6 mutant which through upregulation of p-rps6 downregulated p-Akt causing an increase in the association of Arp3 and its upstream activator N-WASP (neuronal Wiskott–Aldrich syndrome protein) [10]. This was further confirmed using a knockdown of p-Akt by RNAi in SCs which also led to reorganization of actin filaments and BTB restructuring [10]. In the second pathway, the constitutively active quadruple phosphomimetic mutant p-rps6 disrupted insulin/IGF-1 signaling, which inhibited Akt phosphorylation leading to expression of matrix metallopeptidase 9 (MMP-9), a proprotein involved in the proteolysis of tight junction proteins of the BTB contributing for a “leaky” barrier [11]. This was also confirmed using a MMP-9 inhibitor, which effectively blocked the SCs tight junction disruption induced by the active p-rps6 mutant [11]. Importantly, a knockdown of p-Akt using RNAi in SCs resulted in a phenotype identical to the induced by the active p-rps6 mutant causing the SCs tight junction disruption [11]. These findings were recently confirmed by an in vivo study. Using a constitutively active quadruple phosphomimetic mutant to overexpress p-rps6 in vivo, the authors observed a similar phenotype to the previously reported in vitro findings where p-rps6 caused disruption of the BTB, resulting in impaired spermatogenesis due to loss of spermatid polarity and failure in the transport of germ cells [116]. This was a result of p-rps6 induced changes in the spatiotemporal expression of actin and microtubule-based binding and regulatory proteins [116]. In sum, mTORC1 and rps6 signaling control BTB remodeling through changes in actin and microtubule-based binding regulating the transition of preleptotene spermatocytes to the adluminal compartment, and overall spermatogenesis itself.

Interestingly, a recent study by Xiong et al., has suggested a Rheb–mTORC1-independent pathway controlling cell polarity and cytoskeleton organization [117]. Using the Cre–LoxP system to generate two SC-specific mutants (*raptor* and *Rheb* knockout mice), the authors observed that adult *raptor* KO mice displayed azoospermia and disrupted cytoskeletal organization and cell polarity while adult *Rheb* KO mice had intact seminiferous tubules, sperm present in the epididymis and normal fertility [117]. Furthermore, activity of mTORC1 downstream molecules was similar in both models, which suggests that these phenotypic changes were caused by raptor and not by canonical mTOR signaling. In fact, *raptor* but not *Rheb* KO mice had reduced Rac1 activity [118], a GTPase which is part of the Rho family of GTPases, suggesting that this GTPase is involved in raptor-mediated cytoskeletal organization. Whole-transcriptome sequencing revealed that *cingulin*, a gene coding a protein involved in the mediation of interactions between actin and tight junction proteins, was downregulated and even disappeared in some tubules in adult *raptor* but not *Rheb* KO mice [117]. As Rac1 is a GTPase, downregulation could be caused by an increase in GTPase-activating protein (GAP) or a decrease in guanine-nucleotide exchange factors (GEFs). In this case, lower expression of rho guanine nucleotide exchange factor 4 (ARHGEF4), a GEF, was detected [117]. Taken together, these results indicate novel raptor/non-canonical mTORC1 signaling roles for cytoskeleton and cell polarity regulation through the modulation of Rac1 activity by *cingulin*.

Nonetheless, mTORC1 involvement in BTB remodeling is only half of the puzzle. Mounting evidence has shown that rictor, a key component of mTORC2, is also involved in BTB dynamics. Rictor expression is also stage dependent and it is downregulated in late stages, coinciding with BTB restructuring [12]. Studies have shown that *rictor* knockdown by RNAi turns the BTB “leaky” (Figure 3) [12]. In vivo, similar results were observed, as knockdown of *rictor* perturbed BTB integrity due to changes in F-actin organization and a loss of interaction between actin and proteins involved in BTB constitution (α-catenin and ZO-1) [12]. Furthermore, SC-specific amh–Cre-mediated ablation of rictor in mice caused infertility [18]. Loss of rictor also caused microtubule disarrangement and impaired actin organization, which disrupted SC polarity and overall BTB integrity (Figure 3) [18]. These mice had spermatogenic arrest, which supports that mTORC2 is required for BTB integrity. Interestingly, a recent study by Bai et al. explored the possibilities of a conditional germ-cell specific knockout of *rictor* using Ngn3–Cre technology. In this study, *rictor^cko^* mice were also infertile due to impairment of spermatogonial differentiation, which reduced the number of germ cells entering meiosis [107]. Interestingly, loss of rictor also caused apoptosis of early spermatocytes, which further exacerbated this effect. BTB integrity of *rictor^cko^* mice was also compromised due to abnormal localization of BTB components, including basal ectoplasmic specialization and gap junction proteins [107]. Microtubular interactions with actin were also abnormal which disrupted cell–cell junctions and Sertoli–germ cell adhesion [107]. Overall, this study further confirmed mTORC2 involvement in BTB maintenance and suggested new roles for mTORC2 in spermatogonial differentiation, indicating that mTORC1 and mTORC2 overlap, at least partially, in some functions but also have fundamental differences in others. Furthermore, mTORC2 signaling in germ cells seems to orchestrate with SCs to form the correct architecture for a successful spermatogenesis.

Another recent topic of study linking SCs with mTOR has been focused on the metabolic control of these cells by this serine/threonine protein kinase complex. As discussed, SCs are known as “nurse cells” due to their role in providing structural and nutritional support to germ cells [119]. Indeed, these cells also have unique metabolic features, exhibiting a ‘Warburg-like’ metabolism [120] since germ cell metabolism is entirely dependent on SCs that produce the lactate needed as substrate for germ cell development [13]. Thus, the control of SC metabolism is a key event for a correct spermatogenesis. Interestingly, a recent report demonstrated that human SCs exposed to rapamycin had several metabolic parameters altered, including glucose consumption and mitochondrial complex III protein levels [9]. Increased lipid peroxidation and a partial inhibition of mTOR phosphorylation at Ser2448 was also observed in SCs exposed to rapamycin [9]. Finally, phosphorylated 4E-BP1 levels remained unchanged after the treatment which led the authors to speculate regarding a rephosphorylation of this mTOR downstream effector during the treatment [9]. A recent study also reported no alterations in phosphorylated 4E-BP1 levels after rapamycin treatment. However, after exposure to a specific PI3K inhibitor, 4E-BP1 levels were downregulated [98]. These results suggest that rapamycin inhibition of mTOR is not sufficient to inhibit p-4E-BP1, which seems to be directly or indirectly regulated by PI3K. Nevertheless, the mechanisms through which mTOR modulates the SC metabolic state affecting the nutritional support of spermatogenesis remain undisclosed. mTOR dysregulation has also been associated with the establishment of metabolic diseases, including obesity [2]. Several studies have shown the importance of the metabolic state of the individual for a correct spermatogenesis [120,121,122,123]. In fact, subfertility or infertility associated with metabolic diseases has been linked with SC metabolic dysregulation. A recent study reported that treatment of human SCs with glucagon-like peptide-1 (GLP-1) increased p-mTOR levels at Ser244 [124]. GLP-1 analogues are used for the treatment of diabetes and obesity [125] promoting weight loss. Thus, that work suggests novel roles for mTOR in the restoration of fertility in individuals with subfertility or infertility induced by metabolic diseases. However, further studies are required to determine how mTOR signaling is involved and if mTOR is dysregulated in subfertility or infertility cases associated with metabolic diseases.

## 5. Concluding Remarks

Knowledge concerning mTOR indicates that it functions as a master integrator of several upstream signals (amino acids, growth factors, insulin and energy status, among others), which responds accordingly through several downstream effectors. This multiprotein complex is composed by two complexes that share components, mTORC1 and mTORC2, that carry and respond to upstream signals accordingly. Several advancements have been made trying to understand the assembly of mTOR complexes and protein–protein interactions resulting from that process. However, there is still much to be done, particularly in an in vivo environment, which could closely resemble physiological conditions. This subject is of particular importance as only with an exact view of each complex functions and the role of each component in the assembly of mTOR complexes can we fully understand mTOR functions. In fact, there are still components of mTOR complexes whose functions and role are yet to be defined.

mTOR inhibition by rapamycin has been extensively used to better understand mTOR functions. Furthermore, this inhibition has been pursued as a linchpin to better manage several metabolic diseases (including cancer) and the associated co-morbidities. Interestingly, male infertility derived from rapamycin treatment was the first sign of mTOR involvement in male reproduction. Nowadays, several studies have shown different ways of involvement for mTOR in spermatogenesis. However, there is a lack of consensus whether mTOR’s role is positive or negative concerning male reproductive health. As discussed above, several studies in upstream and downstream mTOR effectors present both positive and negative effects concerning SSC maintenance, BTB maintenance/restructuring and overall male fertility. Several studies have also shown that mTOR inhibition is crucial for SSC maintenance. However, mounting evidence in models using knockout of upstream mTOR inhibitors shows that mTOR activation leads to exhaustion of the SSC pool. Different modulators of this mTOR inhibition are also starting to be discovered and some of these modulators are even suggested to be regulated by micro-RNAs. Interestingly, studies have started to show that mTOR activation is stage-dependent, being active in progenitors committed to differentiation. In fact, mTOR transition between active and inactive states also seems to be essential to decide the fate of an SSC. Retinoic acid treatment, a key regulator of spermatogenesis which is involved in spermatogonia differentiation, also resulted in mTOR phosphorylation, and thus also suggests an involvement of mTOR in this process. Taken together, these studies reinforce the deciding role of mTOR in controlling the fate of SSCs.

Another topic of interest is mTOR’s involvement in BTB dynamics. Studies using in vitro and in vivo approaches have shown different actions of mTORC1 and mTORC2 in this barrier. The first is involved in BTB remodeling while the latter is involved in making the BTB “tighter”. As before, mTOR complexes expression is also stage-dependent, which explains the transition of preleptotene spermatocytes to the adluminal compartment due to a timely upregulation of mTORC1 at later stages while mTORC2 is upregulated at earlier stages of the seminiferous epithelial cycle. The attention of the scientific community is now focused on identifying possible signaling pathways regulating this complex interaction and this focus already produced interesting results, identifying the prpS6/Akt/Arp3/N-WASP and the p-rps6/Akt/MMP-9 pathways as mediators of mTORC1 effects in BTB dynamics.

It seems that a small part of the puzzle is starting to be deciphered and that the answer is not what we expected. mTOR seems to be much more than a simple positive or negative trigger in male reproduction. In physiological conditions, it acts as a master integrator of several signals, which is also regulated by different factors in a joint effort to decide the outcome for several processes, including SSC differentiation or self-renewal and BTB restructuring. Nevertheless, these apparently conflicting roles of mTOR in male reproduction underline the complex web of interactions that these multiprotein complexes regulate, which makes the attempt to study them an uphill battle. Trying to translate in vitro results to physiological conditions is also difficult, highlighting the need for more integrative studies that can mimic physiological conditions in order to fully disclose mTOR’s function in male reproductive health. There is no doubt that mTOR’s involvement in male reproduction deserves special merit and attention in the years to come.

## Abbreviations

mTOR	Mammalian target of rapamycin
SSCs	Spermatogonial stem cells
SCs	Sertoli cells
mTORC1	Mammalian target of rapamycin 1
mTORC2	Mammalian target of rapamycin 2
BTB	Blood-testis barrier
raptor	Regulatory associated protein of mTOR
pras40	Proline-rich Akt substrate 40 kDa
deptor	DEP (Dishevelled, Egl-10 and Pleckstrin) domain-containing mTOR-interacting protein
mLST8	Mammalian lethal with sec-13 protein 8
rictor	Rapamycin insensitive companion of mTOR
mSIN1	Mammalian stress-activated protein kinase interacting protein
protor1/2	Protein observed with rictor 1 and 2
FKBP12	FK506-binding protein 12
TSC1/2	Tuberous sclerosis complex
Rheb	Ras homolog enriched in brain GTPase
Akt	Protein kinase B
RSK1	p90 ribosomal S6 kinase 1
ERK	Extracellular signal regulated kinase
PTEN	Phosphatase and tensin homolog
Redd1	Protein regulated in development and DNA damage response 1
PKC	Protein kinase C
Plzf	Promyelocytic leukaemia zinc finger
GNDF	Glial cell-derived neurotrophic factor
FOXOs	Forkhead box proteins
GILZ	Glucocorticoid-induced leucine zipper
USP9X	Spermatogonial deubiquitinase probable ubiquitin carboxyl-terminal hydrolase FAF-X
STRA8	Retinoic acid stimulated gene 8
p-p70s6k	Phosphorylated p70S6 kinase
p-4E-BP1	Phosphorylated eukaryotic initiation factor 4E binding protein 1
PCNA	Proliferating cell nuclear antigen
Dmc1	DNA meiotic recombinase 1
Rps6	Ribosomal protein S6
MSCI	Meiotic sex chromosome inactivation
ZO-1	Zonula occludens-1
N-WASP	Neuronal Wiskott-Aldrich syndrome protein
MMP-9	Matrix metallopeptidase 9
ARHGEF4	Rho guanine nucleotide exchange factor 4
GLP-1	Glucagon-like peptide-1
AMPK	AMP-activated protein kinase
